# Analysis of Competition and Training Videos of Speed Climbing Athletes Using Feature and Human Body Keypoint Detection Algorithms

**DOI:** 10.3390/s22062251

**Published:** 2022-03-14

**Authors:** Dominik Pandurevic, Paweł Draga, Alexander Sutor, Klaus Hochradel

**Affiliations:** Institute of Measurement and Sensor Technology, UMIT—Private University for Health Sciences, Medical Informatics and Technology GmbH, 6060 Hall in Tirol, Austria; pawel.draga@umit-tirol.at (P.D.); alexander.sutor@umit-tirol.at (A.S.); klaus.hochradel@umit-tirol.at (K.H.)

**Keywords:** speed climbing, sports and computer science, movement science, feature detection, neural network, hold detection, multi-person keypoint detection

## Abstract

Compared to 25 years ago, the climbing sport itself has changed dramatically. From a rock climbing modification to a separation in three independent disciplines, the requirements to athletes and trainers increased rapidly. To ensure continuous improvement of the sport itself, the usage of measurement and sensor technology is unavoidable. Especially in the field of the discipline speed climbing, which will be performed as a single discipline at the Olympic Games 2024 in Paris, the current state of the art of movement analysis only consists of video analysis and the benefit of the experience of trainers. Therefore, this paper presents a novel method, which supports trainers and athletes and enables analysis of motion sequences and techniques. Prerecorded video footage is combined with existing feature and human body keypoint detection algorithms and standardized boundary conditions. Therefore, several image processing steps are necessary to convert the recorded movement of different speed climbing athletes to significant parameters for detailed analysis. By studying climbing trials of professional athletes and the used techniques in different sections of the speed climbing wall, the aim among others is to get comparable results and detect mistakes. As a conclusion, the presented method enables powerful analysis of speed climbing training and competition and serves with the aid of a user-friendly designed interface as a support for trainers and athletes for the evaluation of motion sequences.

## 1. Introduction

The attendance at the Olympic Games in Tokyo 2021 forms without any doubt the historical peak in competitive sport climbing. However, the criticized decision of the International Olympic Committee to evaluate a single discipline, which is composed of bouldering, lead climbing, and speed climbing, demands a completely different preparation from the athletes. Compared to the individual and mostly complex movements for bouldering and lead climbing, there are due to the uniformly designed climbing route only a few techniques used in speed climbing. However, this discipline requires high motor skills and addresses different group of muscles leading to a rearrangement of training methods. Therefore, training sessions concentrate on bringing such motion sequences to perfection. Whilst trainers benefit from their experience, it is difficult to detect at first sight mistakes and differentiate two athletes with a similar technique at a competitive race.

Compared to other competitive sports, research in the sport climbing world is underdeveloped, which complicates the evaluation of training and competition results. In general, the research is focusing on sport science topics such as the identification and prevention of injuries or analysis of physiological parameters. While experts are working on the classification of most common injuries [1], certain studies explain noticeable correlations between climbers’ performance and their physique [2].

Common, existing training methods such as tools for the determination of the maximum finger force and related research about its correlation with the degree of difficulty [3,4,5] built a solid base for the measurement of forces while climbing trials. Therefore, several research groups focused on the determination of such forces of static and dynamic motions for the evaluation of multiple parameters [6,7,8]. A Japanese research group combines the measurement of force and momentum with the tracking of the human skeleton by multiple 3D motion capture cameras and body markers for climbing issues [9]. However, due to the high-cost setup of this method, its application is bounded to research proposes.

Most of these drafts are not applicable in the speed climbing discipline. To ensure smooth motion sequences of an athlete focusing just on the execution of various techniques, the usage of non-invasive measurement equipment to prevent physical and psychological restrictions is necessary. One way to achieve such conditions is the development of sensor-embedded climbing holds. For the measurement of contact times while speed climbing, the authors of [10] used touch sensors by Luxov. This commercial system supplies standardized holds for speed climbing route maps based on capacitative effects. On the other side, video analysis of recorded speed races could replace modifications of the climbing wall. For this purpose, Ref. [11] studied recorded runs of the Youth Olympic Games in 2018 with classic video analysis tools and manual marking of specific body points. The precision of such a labeling process is limited and therefore highly bounded to errors. As opposed to this, the analysis of recorded videos in badminton is introduced in [12]. Thereby, the practice of basic skills in this sport enables detecting each player’s motion posture with the aid of the so-called One-Shot learning technique.

A drone-based camera system presented in [13,14] is used for a marker-based motion capturing solution. Therefore, the highly precise tracking of athletes’ body markers enables multiple analysis of 3D parameters. However, this method’s assignment is probably bounded to usage at training and experimental analysis due to its limitation of motion at competitive events. Generally, research in the field of human body estimation in sport science is dedicated and progressive. A systematic review introduced in [15] summarizes the literature of this research topic.

The summary of all the mentioned research projects, especially in the field of speed climbing applications, emphasizes the absence of a simple method for the analysis of speed runs of various athletes excluding any invasive measurement equipment. The following presented method uses only a camera as a measurement unit and its recordings of speed climbing trials as an input for a video analysis tool to compare motion sequences by:

- Position, velocity, and acceleration of the center of gravity (COG);
- Angles of all relevant human body joints;
- Contact time and measured time between two specified holds.

With the aid of these parameters and a user interface designed with and for speed trainers and athletes, the development of the sport itself can be promoted through several analyses of motion and technique. The detection of positive and negative peaks of data sets via comparison of different recordings enables the individual adjustment of training sessions and a suitable preparation for competition. Additionally, the comparison of athletes among themselves by lining up successful video material including, for example, world record trails, allow a physique-optimized perfection of the speed climbing route map.

## 2. Materials and Methods

To enable multiple speed climbing analyses, several image processing steps are necessary. The human body keypoint detection as well as the use of several image analysis algorithms are needed to extract all the obtainable features on every image to get different parameters for the comparison of speed climbing motions. Hence, the following section explains and clarifies different algorithms and will be completed with the introduction of a user interface for trainers and athletes. The recordings from several world cups as well as Olympia published by the International Federation of Sport Climbing (IFSC) are preprocessed and used as an input for the system. Additionally, self-made videos from training units are added to this data set. All of recordings differ from each other by the camera setup following both athletes and focusing on the faster one. Most of the videos were recorded with the common frame rate of 24 FPS and, if not, down-/up-sampled to create comparable conditions. Each side of the speed climbing wall with a height of 15 m, width of 3 m, and overhang inclination of 5${}^{\circ }$ consists of 30 panels at 1.5 m × 1.5 m. Thereon, unitary 20 hand holds and 11 foot holds are fixed at standardized positions on a defined route map.

The aim of the presented method is to get comparable parameters of the climbers without using any additional measurement equipment, which could probably interfere with the athlete’s motion sequences physically or psychologically. Therefore, the comparison of different athletes and recording conditions should be enabled by the usage of various algorithms allowing the contrast of multiple motion-describing parameters within the same scale. The following method describes how to obtain result-relevant values only by the aid of image sequences. Compared to physical sensors, which are mounted on the wall, the holds or the climber himself need to be calibrated, and a preparation of every single sample and the transformation in argumentative units need to be done. Hence, the following subsections are separated:

- The detection of human body keypoints for the isolation of the climber;
- The detection of significant features and their matching frame by frame for comparison of different athletes and competition/recording setups;
- The determination of the camera movement on every sample to get absolute motion sequences;
- The calculation of scale factors for the transformation of the measured data in a world coordinate system.

**Figure 1:**
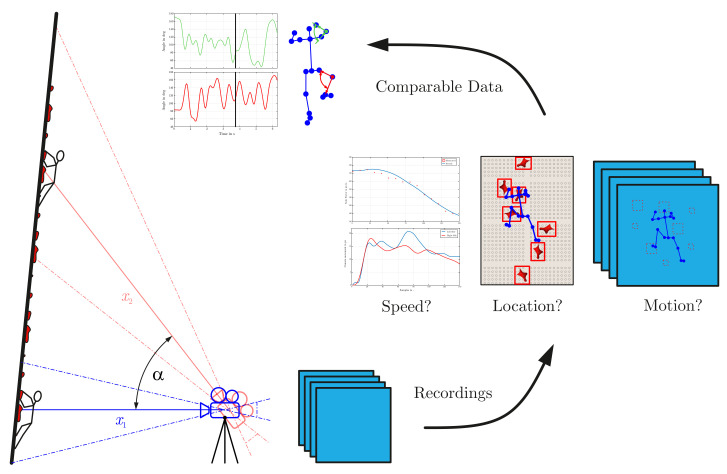
Stepwise illustration of the methodology. Starting from the recording with a specific camera setup, the described method should answer proper questions defining the motion in speed climbing to get a set of data points to compare different athletes.

Hence, the measurement of data for the creation of multiple analysis potentialities is dependent on the usage of several image analyzing steps (see Figure 1).

It should be mentioned that due to the usage of recordings from competitions mainly from a IFSC online database, the camera setup is not consistent for different speed runs. Moreover, parameters such as their height or distance to the wall are unknown but will be estimated with the aid of optimizing algorithms. However, all of the setups, including self-recorded training trials, consist of a single camera placed on a tripod only allowing rotation for the tracking of both athletes. The therewith associated advantages as well as limitations are mentioned in the following sections.

### 2.1. Multi-Person Keypoint Detection

For the calculation of parameters such as joint angles or the position of the COG and its time derivatives, the extraction of the human body skeleton of videos or single images is needed. Therefore, the automated determination of the body keypoints is done by the usage of OpenPose [16]. Based on a convolutional neural network (CNN), OpenPose describes the motion pattern of the recognized athletes by calculating the position of up to 25 body keypoints for every frame. Thereby, in terms of a bottom–up method, image details are determined and associated with body parts with so-called Part Affinity Fields and then linked together to form a larger subsystem until a complete top-level system (corresponding person) is formed. The pretrained system is based on a large dataset composed of the analysis of several recordings of humans in all possible situations and is not explicitly extended with data from specific speed climbing athletes.

The presented method uses only 19 keypoints excluding the ones for the ears, eyes, and small toes.

**Figure 2:**
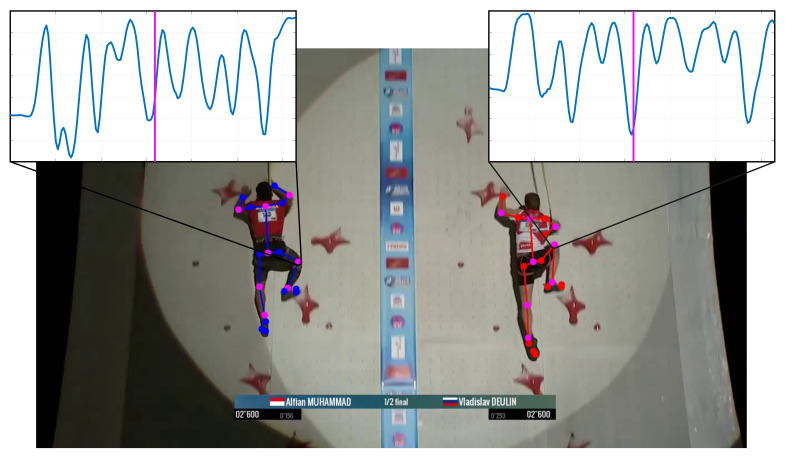
Application of the OpenPose [16] algorithm on a snapshot and the resulting right knee angle distribution of the whole run with the pink line indicating the current frame [17].

Figure 2 shows exemplarily the detected keypoints for both athletes on one recording. The associated algorithm computes then all relevant 2D joint angles over time and COG with the aid of the definition of the human body by the weight and mass center distribution of the single body parts.

### 2.2. Image Analyzing

A key part of this methodology includes the usage of several image processing algorithms such as the segmentation and feature detection followed by a matching section. By using all the available characteristics of every frame, the calculation of world coordinates is enabled and therefore the determination of all needed parameters to compare different athletes and techniques.

#### 2.2.1. Feature Detection and Matching

**Figure 3:**
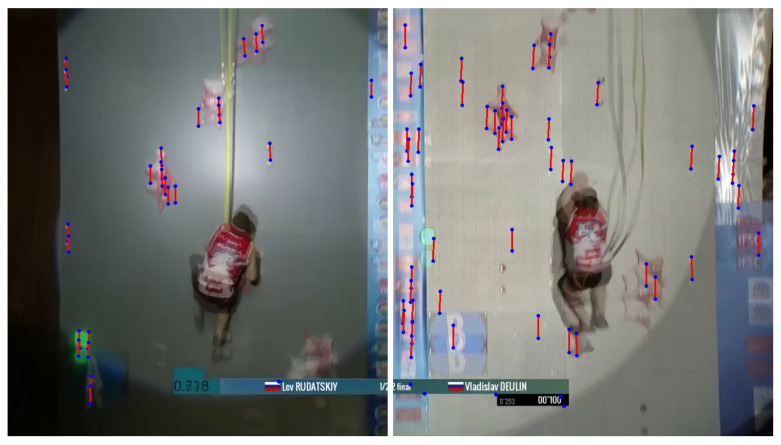
Result of the feature detection and matching pairs algorithm using two consecutive frames for two runs with different environmental conditions [17].

Due to the great variety of recorded videos of speed climbing competitive races, it is important to implement a stable feature detector in a first step. Therefore, the so-called *Greedy Learned Accurate Match Points* (GLAMPoints) algorithm [18] is used to detect relevant high-level feature points frame by frame and additionally match relations of moving objects by comparing frames with their previous ones (see Figure 3).

**Figure 4:**
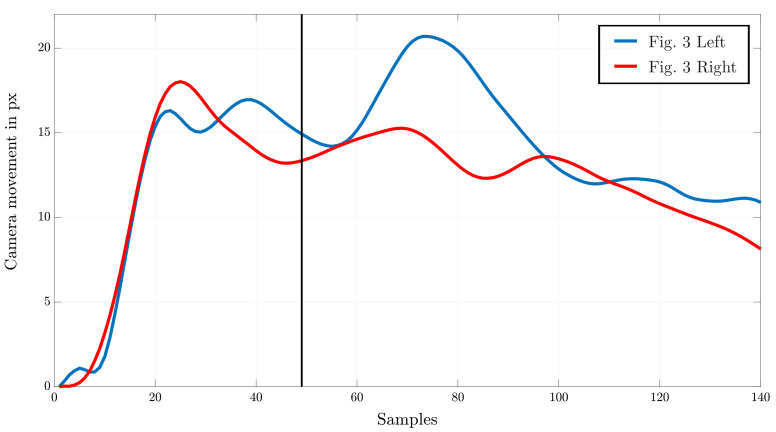
Resulting camera movement in pixels for two different recordings with a vertical line tagging the analyzed frames in Figure 3.

As a result of the varying movement of the climber and recording camera, the resulting feature matches can be used to calculate the shift of static objects and therefore the movement of the camera. In fact, it is unimportant which feature is actually detected and compared. Although the efficiency of the GLAMPoints algorithm finding only correct matching pairs is high, a filtering of false detected points is added by assuming that just pairs with vertical movement are included. As a result, pairs with almost the same distance remain (see Figure 3). Figure 4 reflects the resulting movement of the camera γ after averaging the matched feature point distances for every frame.

The noticeable difference of the presented characteristics in Figure 4 arises through changes in the camera speed due to the focusing behavior of the athletes on every sample. Therefore, the variation of the climbers speed results in an adjustment of the recording, keeping the athlete continuously vertically centered.

#### 2.2.2. Hold Detection

**Figure 5:**
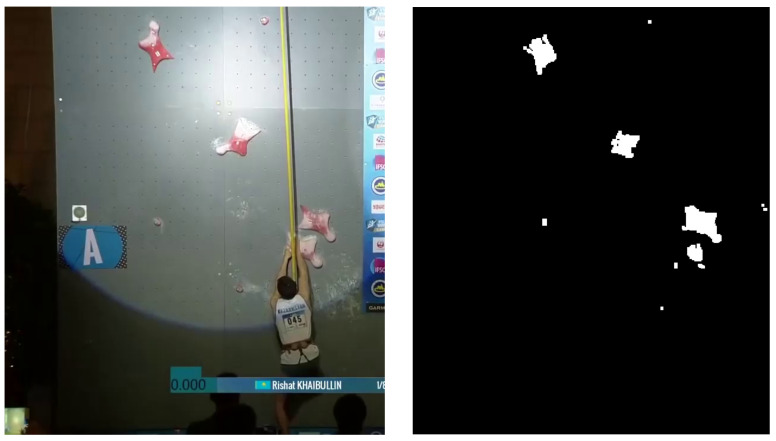
Summarizing figure describing the hold detection algorithm showing the original frame on the left side [17] and the resulting mask with detected areas of the holds on the right side.

The detection of the climbing holds is essential for further calculations due to the identical setup on all walls and therefore builds a base for the comparison of different videos. One possible way to determine the position of all holds is the usage of a color thresholding and region property algorithm illustrated in Figure 5. Involving the fact that all mounted holds on the standardized speed climbing wall are identically and just fixed in various angles, the difficulty detecting them is essentially reduced. Nevertheless, boundary conditions such as the spotlight following the climbers and the chalk covering the majority of most holds call for a transformation from the RGB (Red–Green–Blue) to HSV (Hue–Saturation–Value) colorspace, which allows higher robustness against outliers and therefore increases the efficiency of the algorithm. Hence, by thresholding in a defined, dynamic range and using morphological operations, the remaining regions get labeled and analyzed by e.g., their area or intensity.

Exactly the mentioned boundary conditions, which differ from one video to the other, decrease the possibility of recognizing all holds on every sample and rule the implementation of a consistent detection algorithm out.

**Figure 6:**
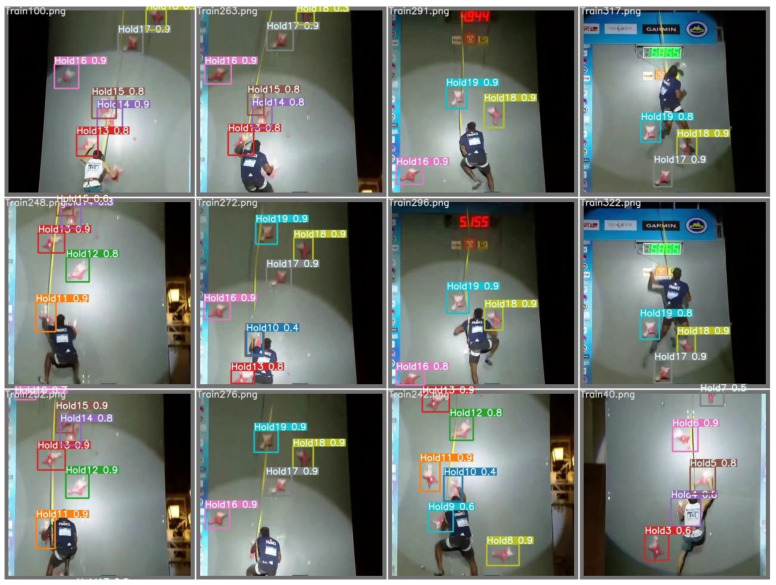
Resulting test batch of a set of images for the detection of speed climbing holds [17].

For that reason, a neural network based on a real-time object detection system is introduced. The pretrained YOLO (You Only Look Once) network [19] predicts the position of the desired objects by its bounding boxes and identifies possible classes. For the detection of specified objects on images, the network needs to be trained with a set of labeled train and test images. The accuracy of this system depends on the amount, quality, and variety of these images. Therefore, 20 videos (720p) of different speed climbing events were analyzed, and all visible climbing holds were manually labeled and classified on over 3000 images (80% training, 20% test frames). Figure 6 demonstrates the training process with analysed test images and the detected holds with label and accuracy from 0 to 1. The resulting network with the calculated weights was subsequently applied on over 400 speed climbing recordings of competition and training to get a big data set for further analysis.

**Figure 7:**
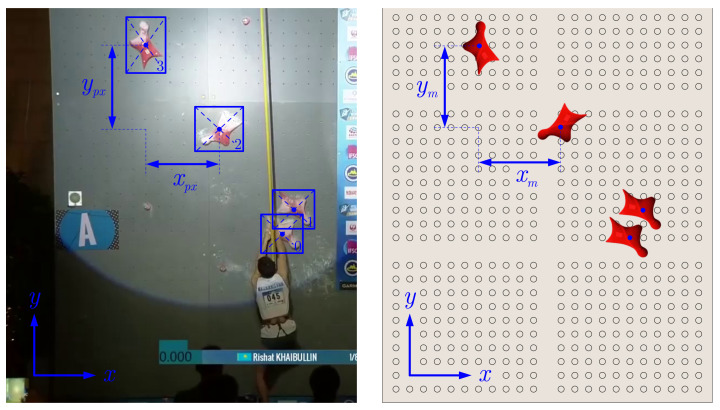
Schematic procedure of the transformation from world coordinates ($x_{m}=0.75$ m, $y_{m}=0.876$ m) to pixels with the actual frame [17] and the cutout of the standardized route map.

Calculating the hold positions of the whole motion sequences helps to improve and extend further analysis. The found unique holds are used for the transformation from pixel values to real-world coordinates. Therefore, the distances in the x- and y-direction from one hold to the next one are matched against the standardized speed climbing wall route map (see Figure 7), which results in an approximate average scale factor for the transformation from pixel values $\left(x_{px}\;,\;\;y_{px}\right)$ to world coordinates $\left(x_{m}\;,\;\;y_{m}\right)$:

(1) $$s\;=\;\frac{1}{2}\left(\frac{x_{px}}{x_{m}}\;+\;\frac{y_{px}}{y_{m}}\right)$$

Since this frame-wise conversion in the x- and y-direction, as has been proven, results in almost the same value, Equation (1) applies both of them for the calculation of their mean.

Ideally, the camera is moving parallel toward the wall while focusing the climber in the center of the current sample. In the case of the most recorded speed climbing videos, the camera is pivoted and placed at a certain height and rotated manually to capture both climbers. Therefore, the transformation of pixel to world coordinates needs to be calculated for every frame.

**Figure 8:**
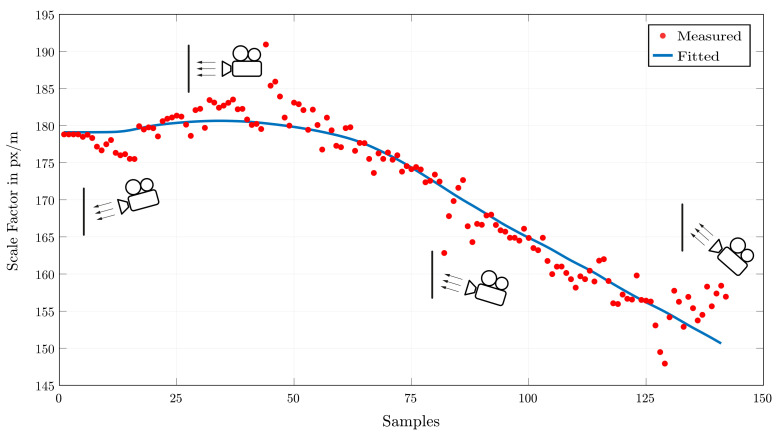
Measured scale factor at some supporting points and the fitted equivalent.

Figure 8 shows such characteristics starting with a slow rise depending on the slight movement of the camera. At the maximum, the distance to the wall is minimized due to a parallel configuration of the camera. With a growing distance based on the vertical rotation of the camera, the scale factor converges to a minimum at the end of the recording.

This progress of the scale value is explained by the consideration of the geometry of such recording setups. Therefore, the used fitting blue line is based on the simplified function

(2) $$s_{i}\;=\;c\cdot \frac{f}{d}\cdot \cos\alpha_{i}\;\;,$$

with *f* (m) as the focal length of the camera and *d* (m) as its distance to the wall. The constant value *c*$\left(\frac{\mathrm{px}}{m}\right)$ is additionally introduced to transform the resulting scale factor to ones with a proper unit. These values are, because of the lack of information on the camera and the setup, unknown and therefore with a least-square means algorithm estimated. The angle $\alpha_{i}$ for every sample *i* is determined by the usage of the previously measured movement of the camera $\gamma_{i}$ and the following iteration:

(3) $$\alpha_{i+1}\;=\;\frac{\gamma_{i}}{c\cdot f}\cdot \cos\alpha_{i}\;+\;\alpha_{i}\;\;.$$

By choosing a start angle of the camera, the relation between camera movement and projecting angle is defined. Using these fitted values serves as an approximation for later calculation and can not be compared to an exact transformation because of the lack of knowledge of particular camera parameters (intrinsic/extrinsic).

Beyond this transformation factor, the detected holds can be used to determine the pasted time between single holds or to synchronize different athletes on different videos at one desired section of the wall. Therefore, the resulting body keypoints, especially the keypoints for the hands and feet, are combined with the hold regions, which describes the moment of touching the selected holds on the speed climbing wall. The following outcome is again used as a subsequent parameter for a comparison between different athletes.

### 2.3. Calculation of Comparable Parameters

With the aid of the calculations described in the previous subsections, the following parameters are determined by the transformation from the image plane to a real-world coordinate system. These parameters again are used for the analysis and comparison of athletes with different techniques and recognition of noticeable mistakes.

In a first step, the resulting positions of all joint keypoints of both athletes are prepared by interpolating missing or bad recognized points over all frames. As a result of the advantageous conditions of the recordings, OpenPose only needs to recognize one athlete for each side, which reduces the number of non-recognition of any body keypoints. Due to the independent detection of both athletes on every frame, the identified body skeleton needs to be assigned to the related side of the speed climbing wall or image, respectively. This beneficial independence is of course required for the separation of both sides and therefore the usage of different video footage of different athletes and competitions and related boundaries. All the presented parameters are subsequently detached and calculated for two chosen athletes.

**Figure 9:**
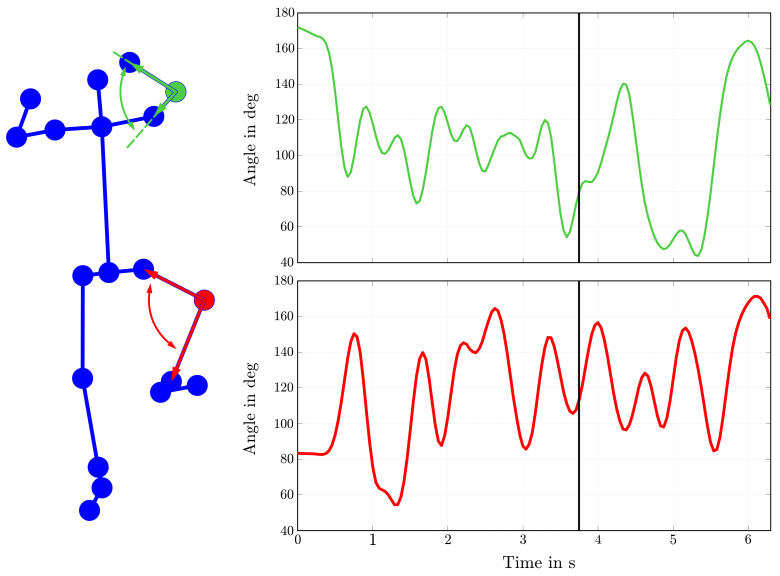
Detected human body skeleton with corresponding angles for right elbow and knee.

An important parameter for the comparison of motion pattern is obviously the angles of relevant joints such as the knee or elbow. Figure 9 shows a constellation of all detected body keypoints, the schematic description of the determination of specific joint angles and their corresponding course over time.

As a result of the analysis of single images, the presented angle results are reduced to a two-dimensional problem. Thus, the computations are done by simple vector calculations and interpolated for missing keypoint detections.

Hence, the gained information about the position of single joints and their angles helps coaches to spot potential conspicuousness at first sight. Additionally, it can be used to compare repeating training sessions of the athletes and, if required, to analyze variety in the quality of other successful athletes with the same or similar technique.

With the aid of the detected keypoints, the COG with its position and velocity can be observed to discuss different movement patterns by separating it in a start, middle, and end section of the climbing wall. Therefore, the position of the COG for every frame $C_{i}$ needs to be calculated by

(4) $$C_{i}\;=\;\sum_{b=1}^{12}m_{b}\cdot \left(x_{b}\cdot r_{b}\;+\;j_{p,b}\right)\;\;,$$

where $x_{b}$ describes the *b*th body part as a vector and $j_{p,b}$ is its proximal joint. Both vectors are determined or identified, respectively, with the aid of OpenPose. The values $m_{b}$ and $r_{b}$ define the particular mass of the related body part and the ratio between the distance of its center of mass and its length. These over several research-estimated parameters can be taken from Table 1 for male athletes.

With the detected location of the COG $C_{i}$, the shift of the camera $\gamma_{i}$, the scaling factor from pixel values to world coordinates $s_{i}$, and the defined frame rate of the recordings φ, the velocity of the COG $v_{i}$ is calculated in the following way:

(5) $$v_{i}\;=\;\left(C_{i+1}\;-\;C_{i}\;+\;\gamma_{i}\right)\cdot \frac{\varphi }{s_{i}}$$

One possible usage of this data set is, as mentioned, the direct comparison of the velocity of each climber in specific sections of the climbing wall. Such results are presented in the following section.

Furthermore, these calculations can be expanded by the determination of the velocity of single body keypoints. The modification of Equation (5) for coordinates of hands and feet enables the calculation of more interesting parameters.

**Figure 10:**
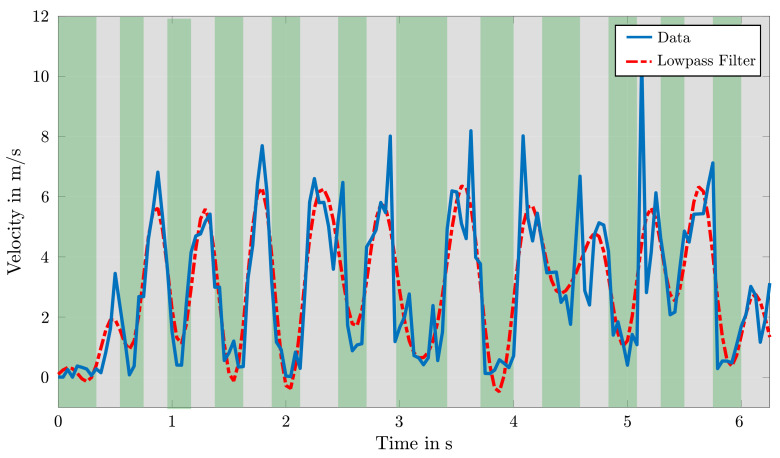
Detection of hold contact times with the velocity data of the right foot with green areas tagging moments of contact.

Figure 10 reflects such resulting distribution of the right foot’s velocity over time. Assuming that the velocity of the corresponding keypoints reaches 0 at the moment of adhering to a hold for a certain amount of time, the detection of contact times becomes simplified by the identification of local minima and neighboring inflection points. Thereby, the inflection points with a negative slope define initial moments of touching specific holds and the ones with positive slopes define the moments of releasing them. For the exclusion of wrong turning points due to detection errors by OpenPose, a low-pass filter is deployed and used for such curve sketching. The related cutoff frequency is thereby determined by analysis of the frequency spectrum.

As a result, the contact time of all touched holds can be used for the comparison of different athletes or for the time wrapping of data sets of athletes with similar motion patterns.

### 2.4. Introduction of a User Interface

The development of a user interface completes the presented methodology by introducing a simple way of its usage for trainers and athletes. Therefore, it combines the detection of the human body skeleton as well as any other features used for the transformation of the inputting frames of the recordings to comparable data sets, as schematically illustrated in Figure 1. Thereby, the user interface implements the following functionalities:

- Reading in single and multiple recordings selected by the user;
- Clipping the read frames on demand for comparison of different record setups;
- Frame-by-frame visualization and analysis;
- Execution of the methodology for the calculation of data sets;
- Illustration of the human body skeleton with all relevant joints and limbs and tracking of the COG;
- Illustration of all detected and numbered climbing holds;
- Export of all determined values.

Additionally, a tool is implemented, which enables the visualization of all exported datasets and the route map of each athlete.

## 3. Results

This section presents the results of two professional athletes with different techniques. It not only demonstrates the amount of data that can be extracted from recordings of competition or training but also the interpretation of the measurements for the detection of possible mistakes and the clarification of the benefits of the declared method. Table 2 shows the exemplary potential of the described algorithms with the enumeration of calculable values for the assembly of a data set for further analysis.

The following data sets originate from the comparison of two athletes with different motion patterns and techniques. Especially the difference of the used technique in the starting section is mentionable. As a result of their success in the speed climbing world, there are among others two unique start motions named by their originators, namely the Reza-Move and Tomoa-Skip. The difference in physique and climbing style leads to significant differences in motion sequences at the first section of the wall. Due to the skip of two holds, athletes using the Tomoa-Start benefit from a shorter start compared to the classic Reza-Move. However, the execution of this technique requires the movement of the left foot in a high position, which leads to a non-ideal knee joint related to a possible speed minima. This significant variation of the movement of the COG in the start section has a great impact on the resulting measurements of the following sections.

**Figure 11:**
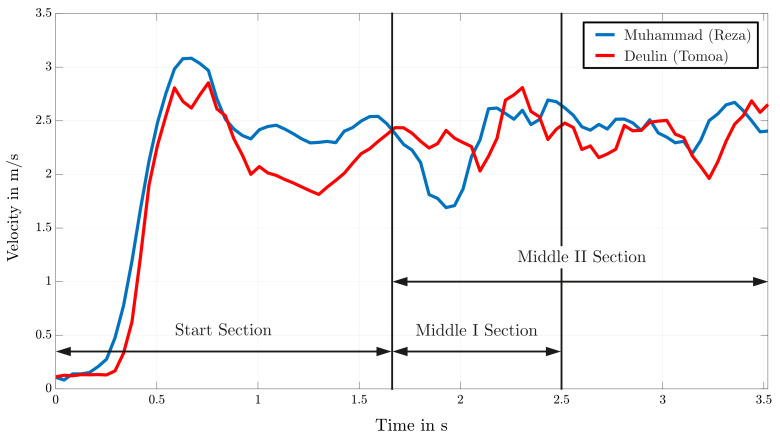
Comparison of the COG’s velocity of two competitive athletes Muhammad (Reza-Move) and Deulin (Tomoa-Skip).

**Figure 12:**
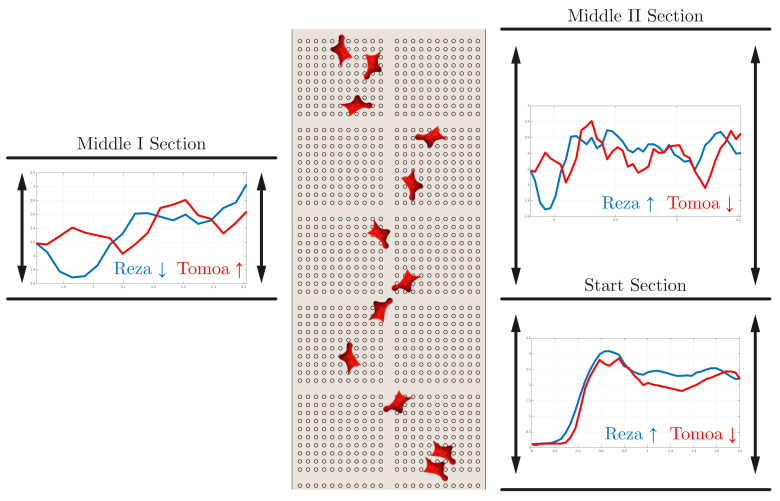
Illustration of the speed climbing wall and the comparison of the athletes’ used technique.

Figure 11 shows the resulting progress of the COG’s velocity over time for the two athletes Muhammad and Deulin for the Start and Middle section of the speed climbing wall. These section-wise comparisons of the velocity enable a more precise analysis of the motion sequences (see Figure 12) and are because of the differences in technique of every single athlete desired. An exact observation of the first third (t≤0.5s) of the start section in Figure 11 approves the disadvantage of the above-mentioned Tomoa-Start with a loss in speed due to the specific movement of the left knee.

**Figure 13:**
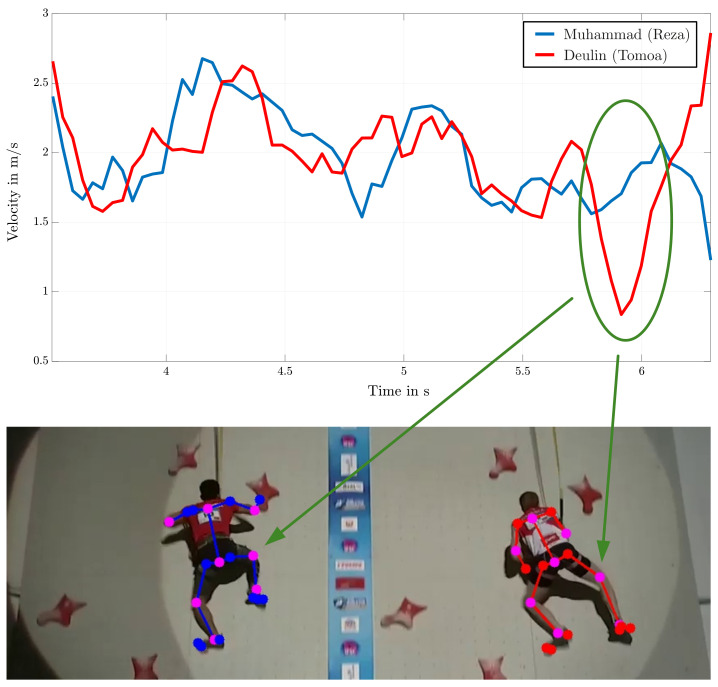
Analysis of the velocity of two athletes in the end section of the speed climbing wall [17].

Generally, analysis of motion in the start and middle sections are done due to the great affect on the outcome at the end. Figure 12 illustrates the separation of the first part of the route map in a Start section with the numbered holds 1 to 5, a Middle I section involving holds 6, 7, and 8, and another Middle II section starting at hold 6 and ending at hold 12. Additionally, the velocities of both athletes with their unique starting technique are shown and by their mean value evaluated and compared. Analyzing these values results in a minor advance for the athlete using the Reza-Move. By evaluating the remaining part of the speed wall and the athlete’s course of the velocity, a huge drop marked in Figure 13 is noticeable. Comparing this with the corresponding sample of the recording leads to a classification of this conspicuity as a mistake. In this case, Deulin’s characteristic movement of the right knee leads to a loss of velocity and time. Instead of pulling the knee to the body to hold the velocity to a constant value, the faulty stretching of this joint triggers this drop and therefore presents a disadvantage compared to his opponent.

Therefore, the advantages in speed shown in Figure 12 as well as the movement mistake presented in Figure 13 explain the outcome of the race (Muhammad Endtime: 5.690 s, Deulin Endtime: 5.799 s). However, not every comparison of two competitive climbers should be done with exclusively speed data. The usage of a big data set composed of parameters among others presented in Table 2 cause a clearer evaluation of the athlete’s performance and a declaration of the result of a speed climbing race.

**Figure 14:**
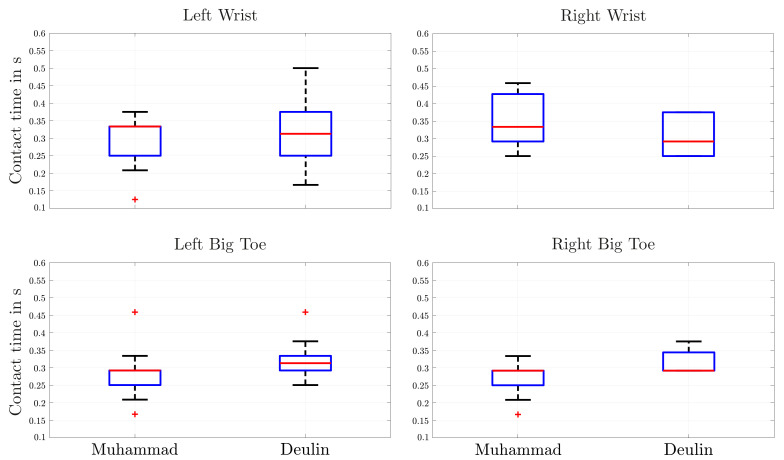
Comparison of contact time of hand and feet keypoints for two different techniques represented in a boxplot.

Not only the analysis of the COG can help to understand the results of a competitive race. In addition, the inclusion of the measurement of contact times of single holds serve as an important aspect for the description of the outcome. Therefore, further analysis of the represented calculation of the velocity of single limbs with Equation 5 and the exemplary determination of contact times in Figure 10 lead to further important parameters for the analysis of the athlete’s motion. On that account, the velocities of hands and feet for both athletes were therewith studied and illustrated by their distributions (see Figure 14).

## 4. Discussion

The methodology describes a way for the determination of significant parameters for the analysis and comparison of motion sequences of speed climbing athletes. As compared to other projects, it uses only the recordings of climbing trials or competitive races as input and forgoes the deployment of any other sensor equipment. With the aid of human body and feature detection algorithms, it breaks every frame down into relevant details, which enables the calculation of comparable values in comprehensible units. With the introduction of a user-friendly interface, the trainers can combine their experience with measured data sets for advanced analysis.

The presented results reflect the potential application of the described method comparing two athletes with different techniques. The separation of the data sets in certain sections of the climbing wall allows more specific analysis and more detailed reflection of the athlete’s motion sequences. The combination of the calculated parameters with the recordings itself enables an additional possibility for the detection of possible mistakes. In addition, the inclusion of the determination of the contact time with the aid of the detected body joints and its computed velocity extended the capabilities comparing different athletes.

However, the methodology still needs to be enhanced and adjusted because of the great variety of recordings and camera setups. The exclusive assignment of recordings as input limits the system to the 2D-coordinate system. As a consequence, the precision depends on the video quality and camera setup. Compared to that, a research group from the USA present a real-time 3D human pose detection algorithm by use of a single RGB camera [21]. Thereby, the described method applies a combination of kinematic skeleton fitting with convolutional neural networks.

Additionally, the detection of the human body keypoints should be carefully examined and therefore the usage of OpenPose questioned. Thereby, the currently applied keypoint detector cannot compete with pose estimation networks such as Darkpose [22,23,24] or AlphaPose [25,26,27] within precision and sample rate. As opposed to these projects focusing on convolutional neural network, a method in [28] was introduced describing the estimation of human body parts combining foreground silhouettes detection with segmentation algorithms including skin tone recognition.

The greatest advantage of the described system is the possibility of the extraction of a great amount of parameters from arbitrary recordings with random camera settings. In contrast, the embedded holds from Luxov used by [10] are limited to the measurement of contact times, whereas the presented algorithms combined with a deployed user interface submit a quick solution for the composition of a big data set for further analysis in the speed climbing sport such as statistical inquiries. The simplicity of the overall system allows the validation of all more or less suitable videos of competition and training and hence the performance evaluation of all known athletes and the identification of the parameters causing success.

Currently, a cooperation with trainers and athletes of the Austrian Climbing Federation is maintained for the continuous validation and advance of the project and the associated software.

## Figures and Tables

**Table 1:** Distribution of weight and mass center ratio for each body segment for males [20].

Body Segment	Mass $m_{i}$ in %	Ratio $r_{i}$ in %
Head	06.94	50.02
Trunk	43.46	44.86
Upper Arm	02.71	57.72
Forearm	02.23	67.51
Upper Leg	14.16	40.95
Lower Leg	04.33	44.59
Foot	01.37	44.15

**Table 2:** An overview of a parameter set calculated by the presented method (based on the center of gravity).

Parameter	Description
Endtime	finishing time (s) of the run
PathSection	path length (m) in the defined section
SpeedMeanSection	speed mean (m/s) in the defined section
SpeedStdSection	standard deviation of speed in the defined section
SpeedSectionEnd	speed (m/s) at the end of the defined section
TimeSection	time (s) required for the defined section
Path	path (m) of the center of gravity of the entire route
Speed	speed (m/s) of the center of gravity of the entire route
ContactHandsMean	mean contact time (s) of hands
ContactHandsStd	std of the contact time of the hands
ContactFeetMean	mean contact time (s) of feet
ContactFeetStd	std of the contact time of feet
SHMean	mean angle between shoulder and hip
SHMaxMin	range of min and max angles between shoulder and hip
SHStd	standard deviation of the angle between shoulder and hip

## Data Availability

Not applicable.

## Abbreviations

The following abbreviations are used in this manuscript:

COG	Center of Gravity
CNN	Convolutional Neural Network
IFSC	International Federation of Sport Climbing
